# Influence of providers and nurses on completion of non-targeted HIV screening in an urgent care setting

**DOI:** 10.1186/1742-6405-11-24

**Published:** 2014-08-04

**Authors:** Rachel A Bender Ignacio, Jacqueline Chu, Melinda C Power, Jeffrey Douaiher, Jordan D Lane, Jeffrey P Collins, Valerie E Stone

**Affiliations:** 1 Division of Allergy and Infectious Diseases, University of Washington, 1959 NE Pacific St, BB545, 98195 Seattle, WA, USA; 2 Department of Medicine, Massachusetts General Hospital, Grey/Bigelow 740, 55 Fruit St, 02114 Boston, MA, USA; 3 Johns Hopkins Bloomberg School of Public Health, Department of Epidemiology, 615 North Wolfe Street, W6508, 21205 Baltimore, MD, USA; 4 Department of Surgery, Johns Hopkins Hospital, 600 N Wolfe St, 21287 Baltimore, MD, USA; 5 Harvard Medical School, Tosteson Medical Education Center, 260 Longwood Ave, 02115 Boston, MA, USA; 6 Department of Medicine, CHC Urgent Care, Massachusetts General Hospital Chelsea Healthcare Center, 151 Everett Ave, 02150 Chelsea, MA, USA; 7 Department of Medicine, Harvard Medical School, 25 Shattuck St, 02115 Boston, MA, USA; 8 Department of Medicine, Mount Auburn Hospital, 330 Mount Auburn Street, 02138 Cambridge, MA, USA; 9 Fred Hutchinson Cancer Research Center, M1B-140 1100 Fairview Ave N, Seattle, WA 98109, USA

**Keywords:** HIV, Screening, Patient care, Urgent care

## Abstract

**Introduction:**

Despite recommendations by the Centers for Disease Control (CDC) that all adults be offered non-targeted HIV screening in all care settings, screening in acute-care settings remains unacceptably low. We performed an observational study to evaluate an HIV screening pilot in an academic-community partnership health center urgent care clinic.

**Methods:**

We collected visit data via encounter forms and demographic and laboratory data from electronic medical records. A post-pilot survey of perceptions of HIV screening was administered to providers and nurses. Multivariable analysis was used to identify factors associated with completion of testing.

**Results:**

Visit provider and triage nurse were highly associated with both acceptance of screening and completion of testing, as were younger age, male gender, and race/ethnicity. 23.5% of patients completed tests, although 36.0% requested screening; time constraints as well as risk perceptions by both the provider and patient were cited as limiting completion of screening. Post-pilot surveys showed mixed support for ongoing HIV screening in this setting by providers and little support by nurses.

**Conclusions:**

Visit provider and triage nurse were strongly associated with acceptance of testing, which may reflect variable opinions of HIV screening in this setting by clinical staff. Among patients accepting screening, visit provider remained strongly associated with completion of testing. Despite longstanding recommendations for non-targeted HIV screening, further changes to improve the testing and results process, as well as provider education and buy-in, are needed to improve screening rates.

## Introduction

At the end of 2008, approximately 236,400 individuals were estimated to have undiagnosed HIV in the United States, representing 20% of the 1.1 million infected individuals [1]. In order to identify these individuals and initiate early treatment of HIV, in 2006 the Centers for Disease Control and Prevention (CDC) recommended that the United States adopt a voluntary, non-targeted opt-out HIV screening strategy for all individuals aged 13-64 years presenting to all health care settings where HIV prevalence is known to be ≥ 0.1% [2]. Attempts at non-targeted screening, while logical in the primary care setting, are hindered by many constraints including stigma and lack of full access to primary care, especially among the most vulnerable populations [3,4]. In the more anonymous and accessible emergency department (ED) setting, adoption of non-targeted screening policies has been limited by time, concerns about follow up of test results, and competing priorities. Previous studies have demonstrated significant increases in detecting undiagnosed HIV with non-targeted, rapid HIV tests in the ED setting [5,6], yet only about 25% of eligible patients complete testing in these studies [3,7-9]. In settings without explicit opt-out screening protocols, there were far lower rates of screening, such as the 1.5% overall screening rate observed within the 2009 National Hospital Ambulatory Medical Care Survey (NHAMCS) data [4]. A recent review of NHAMCS revealed that despite growing recommendations for non-targeted HIV screening in all clinical settings, no increase in screening in EDs had occurred between 1993 and 2010. However, HIV testing was significantly greater in outpatient ambulatory medical care departments (OPDs) than in EDs and physician offices, suggesting that non-targeted screening in urgent care (UC) settings may continue to be an important setting in which to expand HIV testing [4].

UCs may be an opportune setting for HIV screening because physicians may be less constrained by time or by acuity of the patient’s condition compared to the ED setting [10]. At the same time, the UC setting maintains the function of a safety net for patients not linked to primary care who may have had fewer opportunities to be screened for HIV. While a logical and previously studied setting for implementing HIV screening, UCs in prior studies report lower rates of screening than expected. These studies cite the burden of pre- and post-test counseling, inconsistent practice patterns, and workflow as reasons for low screening rates [4,11].

Therefore, we undertook a study of non-targeted HIV screening at Massachusetts General Hospital-Chelsea Urgent Care (MGH-CUC), with the goal of creating infrastructure to streamline the screening process and increase screening rates. The MGH-CUC is a busy urban UC within a large academic-community health center, serving a racially and ethnically diverse underserved patient population. The health center serves a community with disproportionate rates of low health literacy, non-legal immigrant status, and poor social stability. Many of these patient characteristics not only increase the risk of acquiring HIV, but also decrease the likelihood that patients engage in routine primary care. Because no protocols existed for managing results or new HIV diagnoses, the baseline rate of HIV screening at this location was essentially zero. Our goal was to offer HIV screening to all patients by adding a triage questionnaire and testing protocol in the UC, as well as education sessions for all staff. The protocol employed an opt-in testing strategy since restrictions of Massachusetts legislation prior to 2012 required written informed consent for HIV testing (amended to verbal consent on April 27, 2012, which was after completion of this pilot) [12,13]. We examined factors predicting acceptance of HIV screening and completion of testing in this Urgent Care setting.

## Methods

### Study population and clinical pilot

We initiated a four-month pilot to demonstrate feasibility of non-targeted HIV screening in the UC. We performed an observational study augmented with electronic medical record (EMR) review and post-pilot provider and nurse surveys. We attempted enrollment of all patients age 18-65 presenting to the clinic between October 3, 2011 and January 30, 2012. We excluded minors aged 13-17 from the study, despite screening recommendations, because of the added complexity of written informed consent in the State of Massachusetts at the time of the study [12,14]. At triage, the nurses were requested to pair a study packet containing the data collection form (DCF), HIV consent/lab slip, and HIV testing explanatory material with the clinical encounter form for all patients aged 18-65 presenting with any complaint. Nursing staff documented self-report of HIV testing during the last 12 months and patient willingness to participate in HIV screening during that visit. Providers including physicians (MDs), nurse practitioners (NPs), and certified physician assistants (PA-Cs), were requested to offer each patient an HIV test, regardless of responses in triage, and obtain written consent for the test. Following state law, lab slips were not processed unless patient and provider signatures were obtained. Interpreters were used as necessary for the clinical visit. Patients who consented to testing had whole blood from peripheral venipuncture sent for 4^th^ generation EIA/p24Ag HIV test (Abbott ARCHITECT Combo Assay), with positive tests confirmed with reflex Western Blot. Patients were then given additional printed information in multiple languages about HIV with an explanation of how to obtain their results.

For the first 2.5 months of the study, all patients received a provider phone call within 1-3 days of the visit; negative results were given during the call and patients with positive results were asked to return to clinic to receive their results. Interim feedback from providers suggested that calling all patients with their results was neither a successful method for reaching patients and conveying their results nor was it sustainable. For the remaining 1.5 months of the pilot, patients were instructed to obtain their printed results and explanatory information in envelopes held at the clinic’s reception desk. As before, any patients with positive results were called personally and asked to return for a provider visit before the written result was released.

### Provider and nurse surveys

All providers and nursing staff during the 4 months of the pilot were recruited to participate in our post-pilot survey via email. We asked staff to respond to the survey if they had worked a minimum of 1 shift during the pilot period. Staff who chose to participate followed a link to a secure, anonymous survey online. The survey used Likert scales to rate most negative to most positive responses to several statements regarding HIV testing in the Urgent Care Center and included free text for additional comments.

### Data collection

The DCF accompanied the patient throughout the UC encounter. Nurses documented question responses during triage, and providers documented if patients were ultimately offered screening and then consented. If patients either were not offered or did not accept screening, providers documented the reason screening was not completed. Study data were collected and managed using REDCap electronic data capture tools hosted by Partners HealthCare Research Computing, Enterprise Research Infrastructure & Services (ERIS) group. REDCap (Research Electronic Data Capture) is a secure, web-based application designed to support data capture for research studies [15]. Medical record number (MRN) on the DCF was linked with the EMR, providing augmented demographic and clinical data for the patient encounter. HIV testing history back to 1998 was recorded when available from the EMR. The EMR was also used to obtain test results from the pilot and link entries from repeat encounters. DCFs without MRNs were excluded from analysis, as were patients who attended nurse-only visits. Patients with incomplete data due to acuity of visit or provider omission were included in the study and documented as “not completed, lack of documentation/other reason” if a DCF was initiated.

### Data analysis

Data tabulated included self-report of HIV testing within 12 months, age category by 5-year increment, gender, race/ethnicity, Primary Care Physician (PCP) location, area of residence (same city, within clinic catchment, outside catchment area), month of UC visit, prior HIV tests during last 12 months, documentation of HIV test ever in the EMR, triaging RN, and provider seen. We evaluated the univariate relationship between each of these characteristics and completion of screening using Chi-Square tests. We used logistic regression to identify independent predictors of completing screening from the above variables among all participants. We created a second multivariable model restricted to only those patients indicating they desired screening in triage. Repeat visits during the study period were excluded from the primary analysis but included in a repeater analysis. We evaluated the rate of repeat visits in which testing took place both when testing was initially performed and when testing was not initially performed. We report 95% confidence intervals and consider a p-value of <0.05 statistically significant throughout. We performed a qualitative analysis of post-pilot surveys given the small number of surveys. Data was analyzed with SAS, Version 9.3 (Cary, NC, USA).

### Human subjects

Institutional review board (IRB) approval was obtained from the Partners Healthcare System Human Subjects Research Committee for the medical records review and evaluation of the pilot. The pilot itself was considered IRB exempt since it was an innovation of national clinical guidelines. Informed consent was waived for the observational study as consent was obtained routinely for the HIV test. A separate IRB application was submitted and received approval for the provider/nurse survey. Informed consent was implied by response to the survey invitation. The survey recruitment email stated clearly that participation in the survey was optional and responses were free from personal or professional repercussions.

## Results

### HIV screening uptake

**Figure 1:**
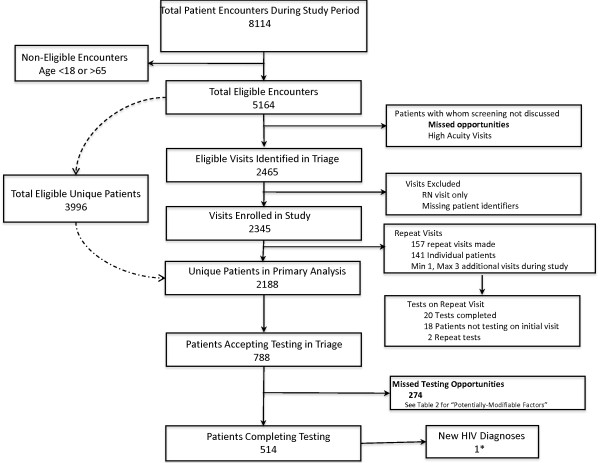
**Patient flow through urgent care clinic during the four month study period.** One new diagnoses resulted from screening. One additional diagnosis resulted from targeted partner testing of the study patient.

Figure 1 illustrates the data collection and HIV screening process. Over the four-month pilot period, 5,164 vistis were made by patients aged 18-65 at the Urgent Care Center out of 8,114 total visits. There were 3,996 unique eligible patients during the pilot period, 8 of whom had previously diagnosed HIV (0.2%). We observed 2,465 eligible visits (47.7%) at which DCFs were initiated. The first visit from each of 2,188 unique patients with eligible visits was included in the primary analysis (after excluding 157 repeat visits and 120 visits where the MRN was missing or the patient was not seen by a provider). The 157 repeat visits made by 141 patients were evaluated in a separate analysis. Of enrolled unique patients, 514 (23.5%) completed HIV screening at their first visit, accounting for only 12.9% of total eligible patients. 788 (36.0%) patients indicated in triage that they were agreeable to screening at their initial enrolled visit. Of 141 patients with 157 repeat visits to the UC during the study period, 17.3% of those not initially screened completed screening on a subsequent visit, while only 5.4% tested more than once during the study period.

One new diagnosis of HIV was made during the screening pilot, with one additional diagnosis made by targeted testing of the partner. There were no indeterminate or false positive tests. Of 514 patients screened, the rate of previously undiagnosed HIV was 0.19%. The newly diagnosed patients were linked to care with an HIV provider within one week at the same health center.

### Patient characteristics

The 2,188 unique patients included in our primary analysis reflected the demographic makeup of the local population [Table 1], with Hispanics predominating (55.7%, mainly of Central American background). The average age was 37 years (SD 12.6 years); 61.6% were male. 1439 patients (65.5%) identified a PCP within the hospital’s primary care network. 49.0% lived within the same city, 40.0% within the adjoining catchment zone of the clinic, and only 11.1% from outside the local area.

**Table 1:** Baseline demographic characteristics of patients presenting to the urgent care during the HIV screening pilot

	**Total sample**	**Accepting HIV screening**	**Tested for HIV**	**Not tested for HIV**
**Variable**	**N=2188**	**N=788 (36.0)**	**N=514 (23.5)**	**N=1674 (76.5)**
Age	37.1 +/- 12.6	34.5+/-11.7	33.8 +/- 11.233.8 +/- 11.2	38.1 +/- 12.8
Male	1342 (61.3)	471 (59.8)	291 (56.6)	1051 (62.8)
Male Gender	1342 (61.3)	471 (59.8)	471 (59.8)	1051 (62.8)
Female Gender	846 (38.7)	317 (40.2)	223 (43.4)	623 (37.2)
**Race/Ethnicity**				
Non-Hispanic White	610 (28.0)	112 (14.2)	61 (11.9)	549 (32.8)
Hispanic	1219 (55.7)	556 (70.6)	373 (72.6)	846 (50.5)
African/Black	127 (5.8)	54 (6.9)	40 (7.8)	87 (5.2)
Asian/Pacific Islander	49 (2.2)	11 (1.4)	6 (1.2)	43 (2.6)
Other/Not Listed	183 (8.4)	55 (7.0)	34 (6.6)	149 (8.9)
**Area of Residence†**				
Same Zip Code	1072 (49.0)	444 (56.4)	307 (59.7)	765 (45.7)
Clinic Catchment	874 (40.0)	281 (35.7)	169 (32.9)	705 (42.1)
Outside Catchment	242 (11.1)	63 (8.0)	38 (7.4)	204 (12.2)
**Pilot Month of Visit Date**				
1	789 (36.1)	323 (41.0)	202 (39.3)	587 (35.1)
2	583 (26.7)	184 (23.4)	131 (25.5)	452 (27.0)
3	397 (18.1)	128 (16.2)	77 (15.0)	320 (19.1)
4	419 (19.5)	153 (19.4)	104 (20.2)	315 (18.8)
**PCP Location**				
At Same Location	989 (45.0)	360 (45.7)	234 (45.5)	750 (44.8)
In System‡	442 (20.5)	134 (17.0)	93 (18.1)	355 (21.1)
No In System PCP	756 (34.6)	294 (37.3)	187 (36.4)	569 (34.0)
**Prior HIV Test by EMR**				
Ever	966 (44.6)		722 (43.1)	248 (48.3)
> 1 year ago	737 (33.4)		541 (32.3)	192 (37.4)
Within 1 year	239 (10.8)		181 (10.8)	56 (10.9)
**Prior HIV Test by Self-Report**				
Within 1 year	565 (25.8)		261 (71.7)	103 (28.3)
**Total Patients**	2188	788 (36.0)	514 (23.5)	1674 (76.5)

Data are No. (percentage) +/- Standard Deviation.

*Abbreviations*: HIV, Human Immunodeficiency Virus; PCP, Primary Care Physician; EMR, Electronic Medical Record.

†Local: same city and zip code as clinic, Clinic Catchment Area: The 5 towns/cities encompassing the 6 zip codes from which >85% of UC population is drawn, Outside Clinic Catchment: a zip code not other than above.

‡In system PCP defined as affiliation with any primary MD or NP affiliated with Partners Healthcare System.

Of patients with a listed in-network PCP, 55.4% had no HIV test documented in our EMR dating back to 1998. 10.8% had tests documented within the last 12 months. There was a large disparity with respect to prior testing by gender, with 53.0% of women and only 30.6% of men having documented prior tests; self-report of HIV screening was more gender-balanced (26.3% of women and 25.1% of men).

### Patient factors associated with completion of screening

Several patient demographic factors were considered in multivariable analysis predicting completion of HIV screening. Younger age was strongly associated with screening acceptance, with screening declining significantly with older age (Odds ratio (OR) 5.2, 95% Confidence Interval (CI) 2.2, 12.2 for patients 18-25 compared against age 60-65, p <0.0001 for trend) [Table 2]. Men screened more frequently than women (OR 1.4, 95% CI 1.1, 1.8). Race/ethnicity also was a significant factor, with Hispanic ethnicity (OR 3.7, 95% CI 2.7, 5.2) or black race (OR 4.1 95% CI 2.5, 6.9) associated with increased rates of screening compared to non-Hispanic white patients (p < 0.0001). There was no statistically significant screening difference between those with a PCP at the health center, those with an in-system PCP, or no listed PCP (p = 0.24). In the study population, 565 (25.8%) reported that they had previously been tested for HIV in the past year; this strongly predicted against screening in this study with an OR 0.3 (95% CI 0.2, 0.3, p < 0.0001).

**Table 2:** Adjusted odds ratios (ORs) and 95% confidence intervals (CIs) for predictors of completing HIV testing from multivariate analysis

	**Full sample (n=2188)**	**Persons accepting HIV screening at triage (n=788)**		
**Characteristic**	**OR (95%CI)**	**p-value**	**OR (95%CI)**	**p-value**
		<0.0001		0.2
18 to 24	5.2 (2.2, 12.2)		5.5 (1.7,17.3)	
25 to 29	3.8 (1.6, 9.1)		4.2 (1.3, 13.6)	
30 to 34	4.9 (2.0, 11.6)		4.8 (1.5, 15.3)	
35 to 39	3.0 (1.2, 7.3)		4.1 (1.2, 13.6)	
40 to 44	2.8 (1.2, 6.9)		4.4 (1.3, 14.8)	
45 to 49	2.6 (1.1, 6.4)		4.3 (1.2, 14.9)	
50 to 54	1.9 (0.8, 4.8)		3.4 (0.9, 12.2)	
55 to 59	1.0 (0.7, 5.0)		2.5 (0.7, 9.7)	
60 to 65	ref		ref	
Gender				
		0.004		0.01
Male	1.4 (1.1, 1.8)		1.6 (1.1, 2.2)	
Female	ref		ref	
Month				
		0.56		0.94
1	ref		ref	
2	0.8 (0.6, 1.1)		1.1 (0.7, 1.8)	
3	0.8 (0.6, 1.2)		0.9 (0.6, 1.6)	
4	0.9 (0.7, 1.3)		1.0 (0.6, 1.7)	
Area of Residence				
		0.08		0.08
Outside Clinic Catchment	0.8 (0.6, 1.0)		0.7 (0.5, 1.0)	
Within Catchment	0.7 (0.4, 1.0)		0.7 (0.4, 1.4)	
Same Zip Code	ref		ref	
Race				
		<0.0001		0.23
Black/African	4.1 (2.5, 6.9)		2.0 (0.9, 4.4)	
Asian/Pacific Islander	1.3 (0.5, 3.2)		0.5 (0.1, 2.1)	
Hispanic	3.7 (2.7, 5.2)		1.5 (0.9, 2.4)	
Other/Unknown	2.0 (1.2, 3.2)		1.2 (0.6, 1.5)	
Non-Hispanic White	ref		ref	
PCP Location				
		0.24		0.44
No In System PCP	1.3 (1.0, 1.7)		1.0 (0.6, 1.5)	
In System PCP	1.1 (0.8, 1.5)		1.3 (0.8, 2.2)	
At Same Location	ref		ref	
Self-Report of HIV Test Within 1 Year				
		<0.0001		0.32
Yes	0.3 (0.2, 0.3)		0.7 (0.4, 1.1)	
Not Reported	0.4, (0.1, 1.2)		1.2 (0.2, 7.3)	
No	ref		ref	
HIV Test in EMR				
		0.008		
Within 1 year	1.6 (1.1, 2.5)		1.1 (0.6, 2.0)	
1 year ago	1.5 (1.1, 1.9)		1.3 (0.8, 1.9)	
Never	ref		ref	
Triage RN				
		0.0005		0.19
A	1.0 (0.5, 2.1)		1.0 (0.4, 2.9)	
B	1.5 (0.5, 4.2)		5.2 (0.5, 49.8)	
C	1.8 (1.0, 3.3)		1.7 (0.7, 4.2)	
D	1.2 (0.6, 2.5)		1.3 (0.4, 3.8)	
E	1.6 (0.9, 2.8)		2.6 (1.1, 5.1)	
F	1.1 (0.8, 1.5)		1.4 (0.9, 2.2)	
G	1.3 (0.6, 2.9)		1.6 (0.5, 5.0)	
H	2.3 (1.4, 3.9)		2.4 (1.2, 5.1)	
I	2.6 (1.7, 4.0)		1.9 (1.0, 3.4)	
J	1.2 (0.2, 6.7)		0.4 (0.1, 2.9)	
K	ref		ref	
Provider				
		<0.0001		<0.0001
A	2.9 (1.6, 5.3)		3.4 (1.6, 7.0)	
B	3.3 (1.8, 6.0)		4.9 (2.1, 11.3)	
C	6.9 (3.3, 14.4)		46.1 (5.5, 390.7)	
D	2.4 (1.2, 4.6)		3.0 (1.2, 7.4)	
E	2.4 (1.0, 5.8)		4.3 (1.1, 16.8)	
F	4.6 (1.9, 11.0)		11.1 (2.1, 60.3)	
G	2.7 (1.3, 5.8)		3.8 (1.2, 12.4)	
H	2.2 (1.2, 4.0)		2.6 (1.3, 5.5)	
I	2.1 (0.6, 7.5)		2.2 (0.4, 11.8)	
J	2.2 (0.9, 5.4)		4.9 (1.1, 21.1)	
K	1.6 (0.6, 4.5)		2.2 (0.5, 9.3)	
L	7.6 (3.4, 17.0)		14.1 (3.4, 58.2)	
M	1.0 (0.2, 5.4)		2.5 (0.2, 32.5)	
N*	1.7 (0.8, 3.4)		1.7 (0.7, 4.3)	
O*	1.6 (0.9, 2.9)		1.5 (0.7, 4.3)	
P*	1.0 (0.5, 2.2)		0.5 (0.2, 1.3)	
Q*	1.6 (0.8, 3.2)		1.6 (0.7, 4.0)	
R*	2.5 (1.2, 5.2)		1.8 (0.7, 4.4)	
S*	3.6 (1.3, 10.4)		10.1 (1.1, 91.2)	
T	ref		ref	

*Abbreviations*: PCP, Primary Care Physician; HIV, Human Immunodeficiency Virus; EMR, Electronic Medical Record.

*Denotes mid-level providers, either certified physicians assistants (PA-C) or nurse practitioners (NP).

In the separate multivariable model restricted to those 788 patients accepting screening, only male gender continued to be a significant patient factor associated with completing the screening test (OR 1.6, 95% CI 1.1, 2.2, p = 0.01).

### Association of provider and nurse on completion of screening

During the four-month pilot period, a staff of 19 clinicians, including Internal Medicine and Medicine-Pediatrics physicians (MDs), NPs, and PA-Cs, provided care, and 11 RNs triaged patients at the UC. In multivariable analysis, the visit provider and the triaging RN were each independent factors associated with completion of screening, contributing more highly in both effect size and level of significance of association (p = 0.0005 for RN, <0.0001 for provider) than many individual patient demographic factors [Table 2 for individual ORs]. There was a 2.6-fold difference in screening rates between those seeing the RNs with the lowest and highest odds of screening completion in the multivariable analysis. There was an 8.7-fold difference in testing rates among providers. In the subset of patients “accepting screening”, the odds of completing screening varied more than 90-fold between providers (p < 0.0001). Point estimates suggest that NPs and PA-Cs tested at lower rates than MDs.

### Acceptance of screening vs completion of HIV test

**Figure 2:**
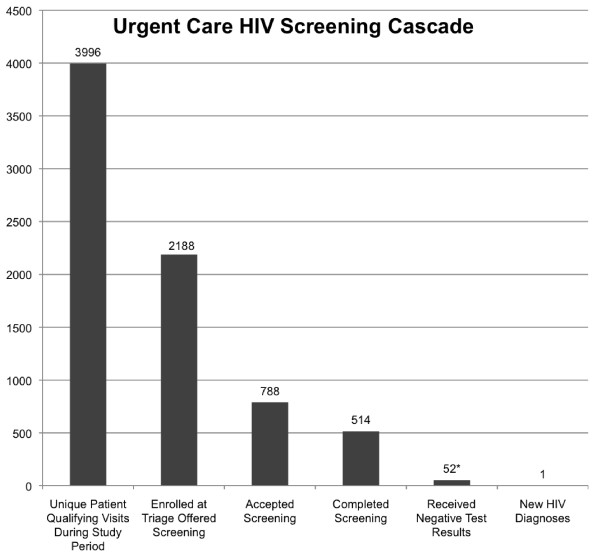
**Cascade of Lost Testing Opportunities.** This cascade shows lost opportunities for screening at each phase in the visit. The largest decrements were in identifying appropriate patients to screen at triage (54.8% of eligible patients identified and enrolled) and obtaining patient acceptance of screening (36.0% of those identified to screen). Of the 788 patients amenable to screening, 34.8% did not complete the consent and blood work. *Approximately 10% returned to receive negative test results. 0.19% of screened patients were found to have HIV.

Starting with all patients age 18-65 eligible for screening, we witnessed a “screening cascade” with drop-offs measured at each phase from entry in the clinic to completion of screening and retrieval of results [Figure 2]. In the flow through the clinic visit, there were clear points where screening opportunities were lost. As described above, 52.3% of eligible visits did initiate a screening questionnaire (DCF) at triage. While 36.0% of patients in those observed visits did accept screening at triage, only 23.5% actually completed the test. 1294 (77.3%) of the 1674 persons not screened had reasons documented that we categorized as visit-based or potentially modifiable factors, such that under different circumstances (less acute visit, different provider) the patient may have accepted and completed screening. Frequently reported modifiable reasons for not completing tested included visit acuity/lack of time (8.1%) or provider not offering testing (8.4%) [See Table 3 for full data]. A small proportion of patients desired HIV screening but provider deferred testing to an upcoming PCP visit (1.2%) or prenatal appointment (1.1%). We included in the “potentially modifiable” list the 552 visits (33%) for which no reason was documented. The majority of these visits had a time-sensitive chief complaint such as chest pain or foreign body in the eye listed in the medical record. An additional 28.1% of visits where screening was not completed were associated with a “patient-based factor”, which we define as a feature of that patient unlikely to change if other visit-based variables were different. Such patient-based reasons included patient report of recent testing in last year (19.8%) or greater than 1 year ago without recent perceived risk (3.5%) or fear of needles (0.24%) [Table 3]. For several encounters, greater than 1 reason was recorded.

**Table 3:** Reported reasons HIV screening not performed during urgent care visit

**Reason screening not performed**	**Number (%)**
**Sums to > 100% with multiple responses**	
Visit-Based or Potentially Modifiable Factors	1294 (77.3)
Provider felt patient to not be at risk	60 (3.6)
Patient felt themselves to be at low risk	367 (21.9)
Provider did not offer	141 (8.4)
Visit Acuity or Lack of Time^†^	135 (8.1)
Patient desired testing, deferred to future PCP visit	20 (1.2)
Pregnancy diagnosed: testing deferred to PNV	8 (1.1)
Patient or provider perceived cost of testing^‡^	7 (0.42)
Patient felt too ill to test	4 (0.24)
Other reason not listed/lack of documentation^†^	552 (33.0)
Patient-Based Factors	471 (28.1)
Patient reported testing in the last 12 months	332 (19.8)
Patient declined, no reason given	68 (4.1)
Patient reported testing > 12 mos prior	59 (3.5)
Previously known HIV+ status	8 (0.48)
Fear of needles	4 (0.24)
Total Patients Not Screened/Enrolled Patients	1674 (100%)

*Abbreviations*: PNV, Pre-Natal Visit.

^†^135 visits documented high-acuity of visit on the study form as reason testing not performed, however EMR review showed that the vast majority of the 552 visits with “Other Reason Not Listed” were also visits of high acuity, but without provider documentation on the study form. Examples of “High-acuity visits” determined by EMR review included presentation with probable cardiac chest pain, asthma exacerbation, foreign body in the eye, or other visit requiring emergent medical interventions and/or ambulance transfer to Emergency Department. Most visits fitting the above description of “High-acuity” also had incomplete HIV screening documentation, presumably because of the urgency of the patient encounter.

^‡^The Commonwealth of Massachusetts offers “universal” health care coverage. For those not already enrolled in the hospital-based safety net system, the vast majority of patients are enrolled following first contact with CHC and covered retroactively, including all lab testing associated with the visit. Despite education regarding this, a small number of providers and/or patients remained concerned about cost of testing.

### Provider and nurse post-pilot surveys

**Figure 3:**
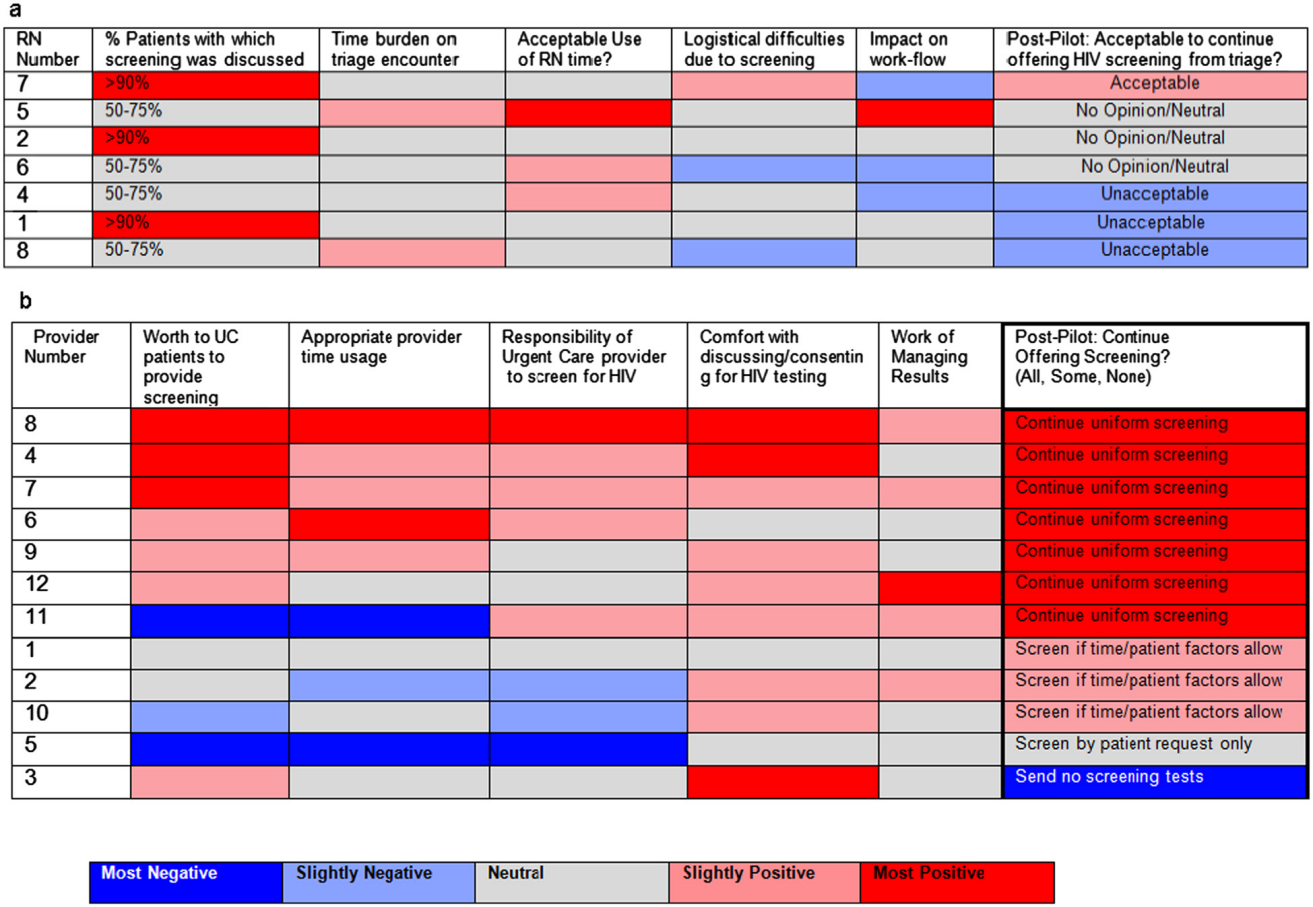
**Heat map representation of post-pilot survey responses a) nursing survey responses.** 7 of 11 eligible RNs responded via secure online anonymous survey. Response scale converted to color coding from “Most Positive” to “Most Negative” response based responses to questions given in the form of Likert Scales as per key. **b)** Provider survey responses. 12 of 19 eligible providers responded via secure online anonymous survey. Response scale converted to color coding from “Most Positive” to “Most Negative” response based responses to questions given in the form of Likert Scales as per key. The final column demonstrates provider self-prediction of their future testing practices after completion of the screening pilot (Screen all patients, only screen some patients depending on patient request/other factors, not perform HIV screening).

Seven of 11 eligible RNs (63.6%) completed a post-pilot survey. Only one RN felt it was acceptable to continue HIV screening after the pilot, 3 had a neutral/no opinion, and 3 were slightly opposed to continue screening. There were less strong patterns with response to other questions and willingness to formalize the screening process; the majority of responses were neutral to slightly negative across the board, especially with respect to logistical difficulties caused by adding screening to the visit and negative impact of work-flow [Figure 3a]. Several open-ended responses from RNs included concerns that it was not appropriate to involve RNs in HIV screening or that RNs did not feel well equipped to discuss patients’ risk of HIV or answer patient questions on this topic.Twelve of 19 eligible providers (63.1%) completed post-pilot surveys with questions that differed slightly from those in the RN survey as appropriate for roll in HIV screening. Of these, 58.3% were in favor of continuing uniform non-targeted screening after completion of the pilot. 25% preferred to screen only a subset of patients, for example, targeted screening based on patient factors and when sufficient visit time allowed. One provider each wished to screen only if the patient requested screening or not at all. Overall, there was a trend in willingness to continue HIV screening following the pilot with positive responses to the following: provider comfort with discussing screening, perceptions of responsibility of UC providers to participate in screening, appropriateness of time use, burden of results management, and value added to patients by offering screening as a service [Figure 3b].

## Discussion

We evaluated predictors of completing non-targeted HIV screening in an Urgent Care center at an academic-community partnership health center serving a racially and ethnically diverse and socioeconomically disadvantaged community. We attempted to enhance the nil HIV screening rate at baseline by establishing a protocol at the UC to offer routine screening. The overall rate of testing at the end of the study was 23.5%, within the range reported by prior studies performed in ED and UC settings [3,7-9]. In the process, we identified a previously unrecognized, independent predictor for completing HIV screening: the health care professional who treated the patient in the UC. Of the responders to the survey, about half of providers were neutral or opposed to the UC setting being the appropriate setting for a screening test, but as illustrated by the heat map of survey responses [Figure 3b], a majority of respondents favored continuing screening. Altogether, provider opinion, whether in favor or opposition to screening, likely contributes to broad range of screening completion rates by provider. This suggests that successful implementation of non-targeted screening is unlikely to occur until there is buy-in by providers in UCs and EDs, despite strong national-level guidelines.

Providers may have offered, completed or deferred screening based on perceived time constraints or because they felt screening was of low value to the patient encounter because of lack of perceived risk, among other factors. While the latter runs counter to the principle of implementing non-targeted testing in acute-care settings, the finding is consistent with previous studies [17,18]. Other studies have demonstrated that physicians have variable knowledge about the CDC recommendations, and when they are informed, often have doubts about guidelines, fear of mistrust from their patients, or fear of what to do with a positive result [17-19].

RN responders to the survey were largely neutral about their role as it related to the screening pilot and were relatively negative with respect to whether they would support non-targeted HIV screening moving forward. In this model of HIV screening, the particular nurse screening a patient could strongly influence the patient’s response as to whether or not he/she was amenable to screening. Since willingness to be screened was evaluated by the triage RN, the group accepting screening was likely selected based on a combination of RN opinion on screening in general, and how the RN did or did not resolve their own perceptions and patient perceptions about who is at risk for HIV.

Based on our results and a recently published study reporting that RN-initiated screening has lower uptake than provider-initiated screening [7], we do not recommend that future non-targeted screening programs use RN-initiated screening protocols in the ED or UC setting. However, because HIV screening inherently involves RNs for other steps in the screening process, it is important to include nursing staff in education and design of HIV screening programs.In this pilot we identified several points during the encounter where improvements could be made to increase both screening uptake and test completion. As depicted in Figure 2, in the flow through the clinic visit, there were clear points where testing opportunities were lost; while there may be several patient factors impacting uptake of screening, our pilot suggests that the person offering the screening is extremely influential in the patient’s testing decision. Not all patients were enrolled in the pilot and offered HIV screening in triage. A lower number of patients than expected were agreeable to screening, possibly reflecting initiation of screening by triage RNs.

Our study also affirmed the previously documented findings that younger age groups, Hispanics, and Blacks were more likely to complete HIV screening [11,16].

### Limitations

Our study has limitations. We performed a single center study, with the intent to evaluate predictors for screening at our site only; patients may have had previous tests performed with outside providers and we relied on their self-report of these tests. Furthermore, a large proportion of visits meeting criteria for enrollment in the study were not enrolled at triage. As the pilot was performed in Massachusetts prior to April 2012, the legal obligation to use written informed consent forced an opt-in strategy. This created an additional barrier to screening that is no longer required by most states. Settings where point-of-care testing is used and where written consent is no longer required may have greater screening uptake.

## Conclusions

The wide variability in completion of screening associated with interacting with different care providers has not previously been reported in the literature. In this single-center observational study we found that the provider and the nurse serving each study participant were each independently associated with acceptance of testing. Based upon our qualitative data, time and comfort level with HIV testing may have contributed to low rates of testing.

While the pilot aimed to educate providers and streamline the screening process, screening drop-off still occurred at points of contact between patients and clinical staff. Survey data suggested that providers maintain bias against uniform non-targeted screening. We can use this knowledge as an opportunity to educate providers on the CDC guidelines and its rationale in order to increase screening in acute care settings. This issue warrants future operational research and clinical innovation projects to enhance the implementation of non-targeted HIV screening across all care settings.

## Abbreviations

HIV: Human immunodeficiency virus; CDC: Centers for disease control and prevention; ED: Emergency department; NHAMCS: National hospital ambulatory medical care survey; OPD: Outpatient ambulatory medical care departments; UC: Urgent care; EMR: Electronic medical record; DCF: Data collection form; MD: Medical doctor, physician; NP: Nurse practitioner; PA: Physicians’ assistant; RN: Registered nurse; IRB: Institutional review board; PCP: Primary care physician; SD: Standard deviation; OR: Odds Ratio; CI: Confidence Interval.

## Competing interests

The authors declare they have no competing interests.

## Authors’ contributions

RBI and JTC designed and conducted the study, interpreted the data and prepared and edited the manuscript. MC provided statistical expertise, interpreted the data and edited the manuscript. JD and JDL assisted with study implementation and preparing the manuscript. JPC assisted with study design. VES provided oversight of study design and manuscript preparation, and edited the manuscript. All authors have read and approve the manuscript.
